# Single-port transvesical enucleation of the prostate (STEP) for benign prostatic hyperplasia: a comparative analysis of patients with large (80–150cc) and very large (>150cc) prostate volumes

**DOI:** 10.1038/s41391-026-01074-y

**Published:** 2026-02-21

**Authors:** Salim K. Younis, Nicolas A. Soputro, Adriana M. Pedraza, Carter D. Mikesell, Abdulrahman Al-Bayati, Samarpit Rai, Lin Wang, Rui M. Bernardino, Ruben Olivares, Mohamed Eltemamy, Zeyad R. Schwen, Riccardo Autorino, Jihad Kaouk

**Affiliations:** Glickman Urological Institute, Cleveland Clinic, Cleveland, OH USA

**Keywords:** Prostatic diseases, Outcomes research

## Abstract

**Introduction:**

Single-Port Transvesical Enucleation of the Prostate (STEP) is a minimally invasive robotic technique for managing benign prostatic hyperplasia (BPH), particularly in prostates ≧ 80 cc. However, its outcomes in very large prostates (>150 cc) remain under-characterized. We compared perioperative and functional outcomes of STEP between large (LP: 80–150 cc) and very large prostates (VLP: >150 cc).

**Materials and methods:**

We retrospectively reviewed 183 patients who underwent STEP using the da Vinci Single-Port system (2019–2024). Patients were stratified into LP and VLP cohorts based on prostate volume (PV). Enucleation efficiency was defined as enucleated tissue weight divided by enucleation time (g/min). Outcomes were compared using descriptive statistics.

**Results:**

A total of 179 patients were included (LP: 93; VLP: 86). Median PV was significantly greater in the VLP group (194 vs. 117 cc, *p* < 0.001). VLP patients had higher PSA (9.7 vs. 6.75 ng/mL, *p* = 0.002), longer enucleation (84 vs. 69 min, *p* = 0.002) and console times (115 vs. 98 min, *p* = 0.010), and improved enucleation efficiency (1.23 vs. 0.97 g/min, *p* = 0.025). Estimated blood loss was slightly higher in VLP (100 vs. 80 mL, *p* = 0.026). All procedures were completed without conversion or additional ports. Hospital stay, catheter duration, and same-day discharge rates were comparable. At 3 months, both groups had equivalent improvements in International Prostate Symptom Score (3 vs. 3, *p* = 0.913), quality-of-life scores (0 vs. 1, *p* = 0.965), and maximum flow rate (17.5 vs. 16 mL/s, *p* = 0.594). Complications were rare, with no readmissions and only two major postoperative events. Urinary continence was preserved in all patients, with transient incontinence occurring in 6 patients (LP: 2; VLP: 4), resolving completely by 3 months.

**Conclusions:**

STEP is effective and scalable for prostates ≥ 80 cc, demonstrating improved enucleation efficiency in very large glands without compromising safety, recovery, or continence preservation.

## Introduction

Management of benign prostatic hyperplasia (BPH) escalates from lifestyle modifications and pharmacologic therapy to surgical intervention. Prostate volume (PV) critically influences surgical approach, complexity, and postoperative outcomes. For large (LP: 80–150 cc) and very large prostates (VLP: >150 cc), current guidelines recommend open simple prostatectomy (OSP), robotic-assisted simple prostatectomy (RASP), and laser-based techniques such as Holmium (HoLEP) and Thulium laser enucleation of the prostate (ThuLEP) [1].

Minimally invasive approaches, particularly robotic platforms, offer superior recovery profiles compared to open surgery. Recent evidence supports advantages of the purpose-built da Vinci single-port (SP) robotic system (Intuitive Surgical, Sunnyvale, CA) over multiport (MP) systems, including shorter hospitalization, reduced postoperative pain, and lower opioid consumption [2–4].

Robotic Single-Port Transvesical Enucleation of the Prostate (STEP), introduced by Kaouk et al. in 2020 [5], employs direct transvesical access to efficiently enucleate adenomas while preserving anatomical structures crucial for continence, including the external urinary sphincter and bladder neck. Initial reports demonstrate minimal blood loss, low complication rates, reduced analgesic needs, and high rates of same-day discharge [6–9]. STEP has also shown lower transient urinary incontinence (TUI) rates compared to HoLEP, with comparable perioperative outcomes [10], and allows concurrent management of bladder stones and diverticula [11].

Despite promising initial outcomes, STEP performance across different PV categories, especially very large glands (>150 cc), remains inadequately characterized [12]. This study evaluates STEP outcomes stratified by PV, assessing its scalability, safety, and efficacy across clinically significant thresholds.

## Materials & methods

### Patient population and selection criteria

A retrospective analysis of a prospectively maintained Institutional Review Board-approved database was conducted to identify all consecutive STEP procedures performed by three surgeons at a single academic institution between February 2019 and August 2024. Patients who met the inclusion criteria presented with PV ≥ 80 cc complaining of lower urinary tract symptoms (LUTS) refractory to medical therapy (5-alpha reductase inhibitor and/or alpha-blocker) or those complicated by indwelling Foley catheterization, urinary retention, recurrent UTIs, bladder stones, hematuria, or hydronephrosis. Patients with a PV < 80 cc were excluded from the analysis.

### Surgical technique

**Fig. 1:**
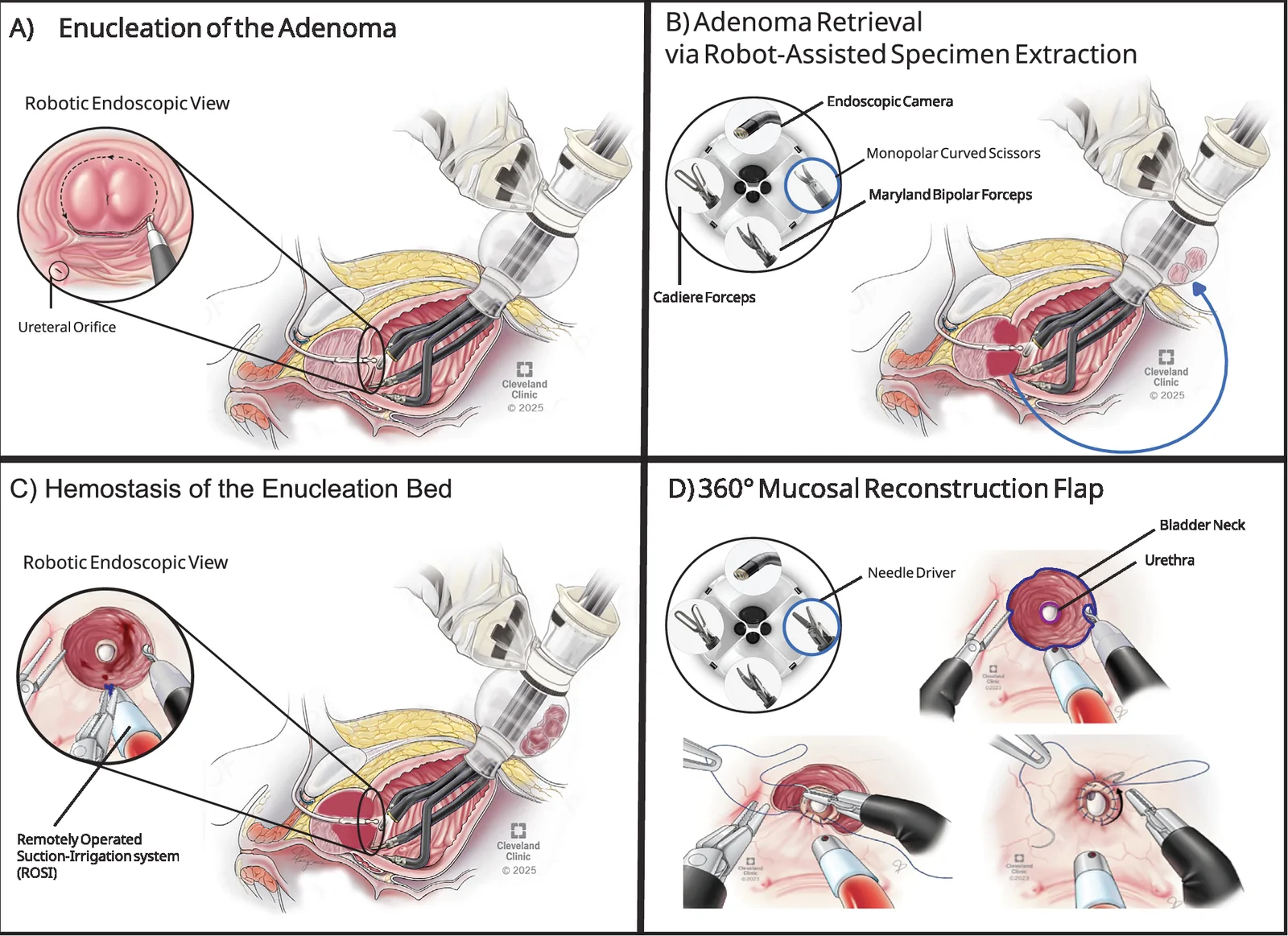
Single-port transvesical enucleation of the prostate surgical technique. **A** Intravesical endoscopic view of the enlarged prostate showing the initial circumferential mucosal incision (dashed line) delineating the enucleation plane. **B** Robotic Cadiere forceps retrieving enucleated adenoma fragments and depositing them into the SP access port bubble chamber for subsequent extraction. **C** Hemostasis of the enucleation bed using the remotely operated suction irrigation system (ROSI) combined with monopolar coagulation under direct magnified vision. **D** 360° mucosal reconstruction flap: the bladder neck is approximated to the urethral stump using a needle driver (ND) and barbed suture to optimize hemostasis.

All procedures were performed using the da Vinci SP robotic system, as previously described by our group and demonstrated in Fig. 1 [13]. Patients were placed supine on a flat operating table. After sterile preparation and draping of the lower abdomen, a 3-cm midline suprapubic incision was made for transvesical access. The anterior rectus fascia was opened, the bladder was identified, and a cystostomy was performed. The robot was docked with instruments inserted through the da Vinci SP Access Port kit (Intuitive Surgical, Sunnyvale, CA), using a floating docking technique [14] and an insufflated bubble port. Pneumovesical pressure was maintained below 12 mmHg. The trigone and ureteral orifices were identified, and a semicircular incision was made in the posterior bladder mucosa, using the prostatic surgical capsule as a landmark (Fig. 1a). Enucleation was initiated at the median lobe, when present, followed by the lateral lobes, from base to apex. Enucleated adenoma was retrieved via robotic arm extraction and placed in the bubble port (Fig. 1b). The verumontanum was used as the distal landmark for excision. Reconstruction involved creating a 360° bladder mucosal advancement flap, which was sutured circumferentially to the urethra using 3-0 barbed sutures, beginning posteriorly and progressing clockwise to the 12 o’clock position [15].

### Variable definitions

Preoperative data included age, body mass index (BMI), Charlson Comorbidity Index (CCI), and prior prostate interventions. PV was primarily measured by magnetic resonance imaging (MRI). When MRI was unavailable or when cross sectional imaging had already been obtained for other indications, PV was estimated using computed tomography (CT) or transrectal ultrasound (TRUS) to support operative planning. Functional assessments included the International Prostate Symptom Score (IPSS), quality of life (QoL) score, Sexual Health Inventory for Men (SHIM), serum prostate-specific antigen (PSA, ng/mL), maximum urinary flow rate (Qmax, mL/s), and post-void residual volume (PVR, mL).

Intraoperative variables included robotic console time (subdivided into enucleation and reconstruction phases), estimated blood loss (EBL, mL), use of surgical drains or continuous bladder irrigation (CBI), Foley catheter duration (days), and intraoperative complications. Enucleation efficiency was calculated as postoperative adenoma weight (g) divided by enucleation time (min). The percentage of prostate adenoma removed (%PAR) was defined as specimen weight normalized to preoperative PV. Surgical versatility was assessed by documenting management of concurrent pathology (e.g., bladder stones or diverticula). All surgical specimens were reviewed by genitourinary pathologists to identify incidental prostate adenocarcinoma.

Perioperative variables included hospital length of stay (LOS, hours), rate of same-day discharge (defined as patients scheduled and discharged on the same day), opioid use, and follow-up attendance. Postoperative assessments included PSA, IPSS, QoL, SHIM, Qmax, and PVR. Complications occurring within 90 days were classified using the Clavien-Dindo (CD) system [16]. Transient urinary incontinence (TUI) was defined as the use of ≥2 pads/day with symptom resolution within 3 months, while persistent urinary incontinence (UI) was defined as incontinence lasting ≥3 months. Patients with preoperative UI or prior BPH procedures were excluded from continence analysis. Treatment-related change (Δ) was calculated as the difference between preoperative and postoperative values for each clinical parameter.

### Statistical analysis

Patients were stratified into two groups based on PV, with a cutoff of 150 cc, in alignment with AUA guidelines for surgical treatment stratification [1]. Categorical variables were summarized as frequencies and proportions (%), while continuous variables were reported as medians with interquartile ranges (IQR). Statistical analyses included chi-square or Fisher’s exact tests for categorical variables and independent sample *t*-tests for continuous variables, as appropriate. Linear regression was used to evaluate associations between prostate volume and operative parameters. To assess whether the learning curve influenced enucleation efficiency, we fit a multivariable linear regression including prostate volume, the number of prior STEP cases for the operating surgeon (continuous), and surgeon as covariates, with an interaction term between prostate volume and prior cases. Case sequence was additionally dichotomized at the median number of prior cases to generate early (≤median) and late (>median) experience groups for subgroup visualization. All analyses were conducted using R software (version 4.4.2; The R Foundation for Statistical Computing, Vienna, Austria), with statistical significance defined as *p* < 0.05.

## Results

A total of 183 consecutive STEP procedures were identified, with no conversions to open surgery or additional port placement. Four patients were excluded due to PV < 80 cc. The final analytic cohort included 179 patients, stratified into large prostate (LP: 80–150 cc; *n* = 93) and very large prostate (VLP: >150 cc; *n* = 86) groups. Median PV was 150 cc (range: 81–425 cc). Baseline demographic and clinical characteristics were comparable between groups, including age (*p* = 0.117), BMI (*p* = 0.225), CCI (*p* = 0.082), and prior BPH interventions (*p* = 1.000). Preoperative functional assessments, including IPSS, QoL, Qmax, and PVR did not differ significantly between cohorts (Table 1).

**Table 1:** Baseline clinicodemographic characteristics, preoperative symptomatic status, and intraoperative parameters.

	Total cohort (179)		T-test and Chi square
Variables	LP	VLP	*p*-value
	(*n* = 93)	(*n* = 86)	
Clinicodemographic			
Age (years), median (IQR)	70.4 (64.4–74.2)	71.4 (66.6–76.4)	0.117
BMI (kg/m^2^), median (IQR)	28.2 (25.7–31)	28.2 (24.8–32.6)	0.225
CCI, median (IQR)	3 (2–4)	3 (3–5)	0.082
Race (Caucasian), *n* (%)	80 (86%)	66 (76.7%)	0.11
Anticoagulation, *n* (%)	10 (10.75%)	7 (8.14%)	0.617
Previous abdominal surgery, *n* (%)	40 (43%)	38 (44.7%)	0.91
Previous prostate interventions, *n* (%)	20 (22%)	19 (22%)	1
Indwelling Foley catheter, *n* (%)	31 (35.6%)	40 (48.78%)	0.097
Prostate volume and preoperative functional status			
Prostate volume (cc), median (IQR)	117 (100–134)	194 (169–235)	**<0.001**
PSA (ng/ml), median (IQR)	6.75 (3.6–10.2)	9.7 (4.8–15)	**0.002**
IPSS, median (IQR)	18 (13–24)	23 (13–26)	0.175
QoL, median (IQR)	4 (3–5)	4 (3–5)	0.643
Qmax (mL/s), median (IQR)	6 (4–9)	5.5 (4–7)	0.959
PVR (mL), median (IQR)	118 (50–350)	122 (72–366)	0.686
SHIM, median (IQR)	18 (6–22)	14 (5–19)	0.375
Imaging, cystoscopy, and operative findings			
Median lobe, *n* (%)	63 (67.7%)	57 (66.3%)	0.835
Bladder stones, *n* (%)	18 (22.8%)	16 (18.6%)	0.993
Diverticulum, *n* (%)	7 (12%)	9 (18%)	0.267
Intraoperative outcomes			
Console time (minutes), median (IQR)	98 (70–120)	115 (82–143)	**0.01**
Enucleation time, median (IQR)	69 (48–87)	84 (62–117)	**0.002**
Reconstruction time, median (IQR)	25 (21–32)	27 (20–35)	0.325
Enucleation efficiency (g/min), median (IQR)	0.97 (0.73–1.5)	1.23 (0.9–1.8)	**0.025**
Specimen weight (g), median (IQR)	62 (41–78)	108 (83–137)	**<0.001**
Percentage of adenoma removed, median (IQR)	52.1 (39.65–64.4)	55.6 (43–66.3)	0.201
Estimated blood loss (mL), median (IQR)	80 (50–100)	100 (53–150)	**0.026**
Intraoperative complication, *n* (%)	2 (2%)	1 (1.18%)	1
Continuous bladder irrigation (CBI), *n* (%)	2 (2%)	0 (0%)	0.499
Surgical drain placement, *n* (%)	0 (0%)	1 (1.16%)	1
Concurrent urological procedures, *n* (%)	18 (19.4%)	19 (22%)	0.6513

*IQR* Interquartile Range, *BMI* Body Mass Index, *CCI* Charlson Comorbidity Index, *PSA* Prostate Specific Antigen, *IPSS* International Prostate Symptom Score, *QoL* Quality of life, *Q*max Maximum flow rate, *PVR* Post-void residual, *SHIM* Sexual Health Inventory for Men questionnaire.

Bold values indicate statistically significant differences between groups based on two-sided testing (*p* 0.05).

**Fig. 2:**
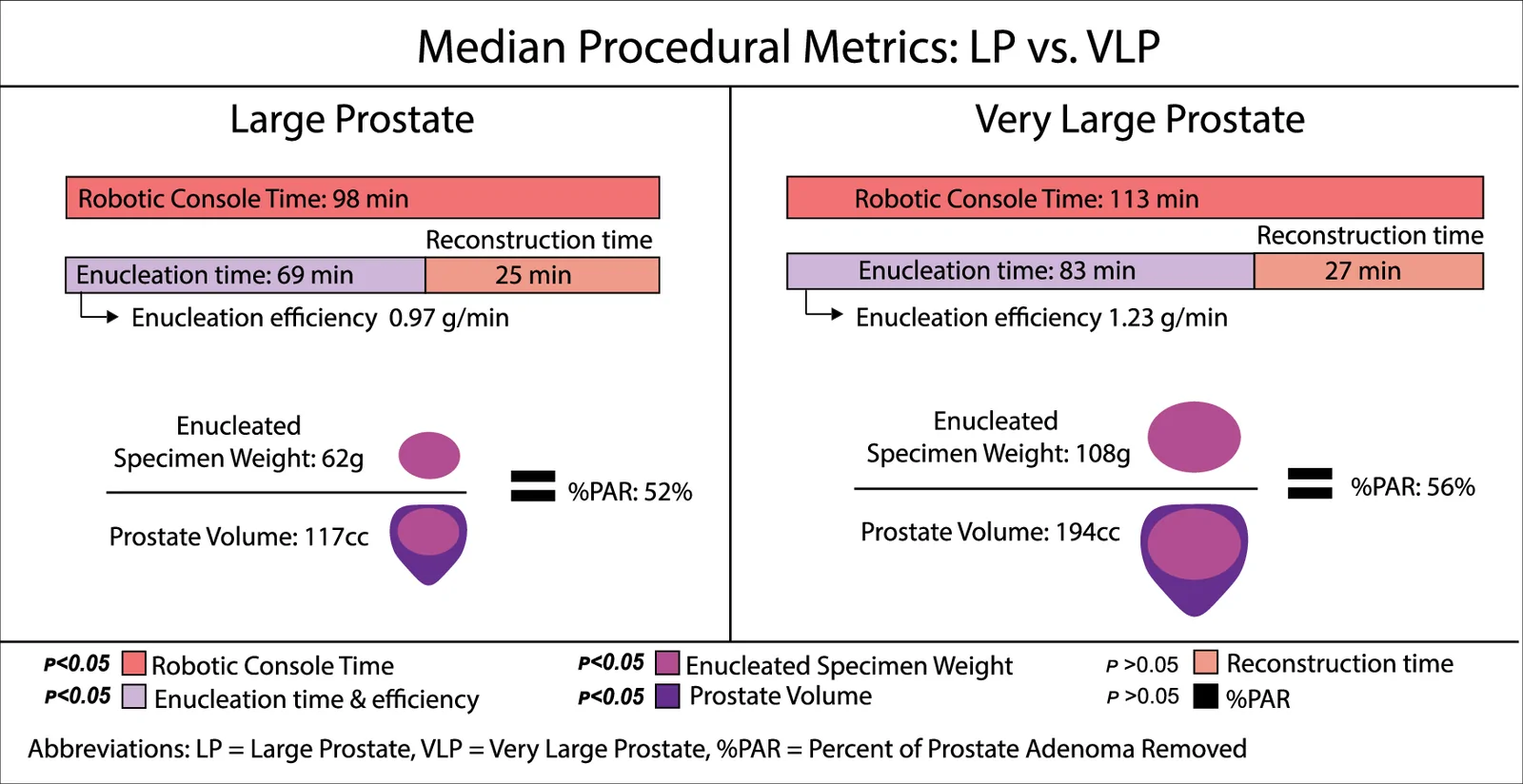
Median procedural metrics by prostate size group (LP vs. VLP). Bar graph displays console time, enucleation time, reconstruction time, and enucleation efficiency. The schematic below illustrates the proportion of adenoma removed (light purple) relative to total prostate volume (dark purple). Significant associations (*p* < 0.05) were observed for console time, enucleation time, enucleation efficiency, specimen weight, and EBL; reconstruction time and %PAR were not statistically significant.

Operative metrics are summarized in Table 1. Console time was longer in the VLP group (115 vs. 98 min; *p* = 0.010), attributable to increased enucleation time (84 vs. 69 min; *p* = 0.002), while reconstruction time remained comparable (27 vs. 25 min; *p* = 0.325). Despite longer operative duration, enucleation efficiency was significantly higher in the VLP group (1.23 vs. 0.97 g/min; *p* = 0.025). Specimen weights were greater in VLP patients (108 vs. 62 g; *p* < 0.001), but the %PAR was similar between groups (55.6% vs. 52.1%; *p* = 0.201) (Fig. 2). Linear regression analysis confirmed that increasing PV correlated significantly with enucleation time (Regression Coefficient (RC): 0.28; 95% CI: 0.13–0.42; *p* < 0.001) and enucleation efficiency (RC: 0.005; 95% CI: 0.002–0.007; *p* < 0.001). No significant associations were observed with reconstruction time (RC: 0.006; *p* = 0.786) or %PAR (RC: 0.034; *p* = 0.120) (Table 2).

**Table 2:** Linear regression analysis to assess direct correlation between prostate volume and procedural outcomes.

Variable	Regression coefficient	95% Confidence Interval	*p* value
Console time (minute)	0.26	0.093–0.419	**0.003**
Enucleation time (minute)	0.28	0.130–0.421	**<0.001**
Enucleation efficiency (g/min)	0.005	0.002–0.007	**<0.001**
Reconstruction time (minute)	0.006	(−0.037)–0.049	0.786
%PAR (g/cc)	0.034	(−0.009)–0.076	0.12
Specimen weight (g)	0.596	0.525–0.666	**<0.001**
EBL (mL)	0.474	0.252–0.696	**<0.001**

*%PAR* Percentage of prostate adenoma removed, *EBL* Estimated blood loss.

Bold values indicate statistically significant differences between groups based on two-sided testing (*p* 0.05).

On multivariable linear regression controlling for surgeon and number of prior cases, PV remained an independent predictor of higher enucleation efficiency (RC = 0.0038 ± 0.0009, *p* < 0.001), whereas experience itself was not significant (RC = –0.0018, *p* = 0.46). The interaction between PV and experience was also non-significant (*p* = 0.77). When stratified into early (≤median) and late (>median) experience groups, both subgroups demonstrated similar positive correlations between prostate volume and efficiency (*p* = 0.78 for difference in slopes).

Concurrent procedures were performed in 18 LP patients (19.4%) and 19 VLP patients (22.0%; *p* = 0.651), including cystolithotomy (*n* = 14 in each group) and diverticulectomy (*n* = 1 in each group). EBL was slightly higher in the VLP group (100 vs. 80 mL; *p* = 0.026); however, only one VLP patient (PV: 365 cc) required transfusion (1.2%; *p* = 0.480). Intraoperative complications occurred in three patients (2 LP, 1 VLP), all involving venous air embolism during early cases. No further complications were observed following technique modifications.

Postoperative outcomes are detailed in Table 3. Median hospital LOS was similar between groups (4.6 vs. 5.0 h; *p* = 0.171), with same-day discharge achieved in 91% of LP and 84% of VLP patients (*p* = 0.282). Most patients were discharged without opioid prescriptions (62% LP vs. 85% VLP; *p* = 0.181). Foley catheter duration was identical in both groups (5 days; *p* = 0.900). Major complications (CD ≥ III) occurred in one patient per group (1.0% LP vs. 1.2% VLP; *p* = 1.000), both due to clot retention requiring cystoscopic evacuation, with no hospital readmissions.

**Table 3:** Perioperative outcomes, 90-day complications, and functional outcomes.

Variables	LP	VLP	*p*-value
	(*n* = 93)	(*n* = 86)	
Perioperative outcomes			
Length of stay (hours), median (IQR)	4.6 (3.7–5.9)	5 (3.8–6)	0.171
Outpatient procedure, *n* (%)	60 (91%)	48 (84.2%)	0.282
Incidental adenocarcinoma, *n* (%)	4 (4.3%)	2 (2.3%)	0.684
Days with Foley Catheter, median (IQR)	5 (4–7)	5 (3–7)	0.888
Opioid-free at discharge, *n* (%)	58 (62.4%)	73 (85%)	0.181
Blood transfusions, *n* (%)	0 (0%)	1 (1.2%)	0.48
90-day major postoperative complications, *n* (%)	1 (1%)	1 (1.2%)	1
90 days hospital readmission, *n* (%)	0 (0%)	0 (0%)	1
Failed trial of void, *n* (%)	2 (2.2%)	1 (1.23%)	1
Functional outcomes			
Time to follow-up (months) included in follow-up analysis, median (IQR)	3.9 (3–7.1)	3.6 (3.1–6.3)	0.716
Time to last follow-up (months), median (IQR)	6.1 (3.3–12.2)	7.5 (3.4–12.4)	0.777
PSA (ng/mL), median (IQR)	0.87 (0.34–2.5)	0.78 (0.5–2.1)	0.61
△PSA (ng/mL), median (IQR)	5.25 (2.8–8.2)	8.8 (4.4–19)	**0.003**
Postoperative IPSS score, median (IQR)	3 (1–6)	3 (1–7)	0.913
△IPSS score, median (IQR)	14 (8–17)	13 (8–21)	0.336
Postoperative QoL score, median (IQR)	0 (0–1)	1 (0–1)	0.965
△QoL score, median (IQR)	3 (2–5)	3 (2–5)	0.626
Postoperative Qmax (mL/s), median (IQR)	17.5 (11–27)	16 (10–25)	0.594
△Qmax (mL/s), median (IQR)	−5 ((−9)–(−0.6))	−12 ((−19)–(−6))	0.141
Postoperative PVR (mL), median (IQR)	12.5 (0–33)	19 (0–54)	0.288
△PVR (mL), median (IQR)	70 (0–388)	82 (21–231)	0.717
Postoperative SHIM score, median (IQR)	14 (5–21)	13 (5–16)	0.477
△SHIM score, median (IQR)	0 ((−1)–(6))	0 ((−1)–(1))	0.421
Urinary continence status			
Transient urinary incontinence, *n* (%)	2 (3.4%)	4 (7.5%)	0.272
Urge, *n*	0	4	
Stress, *n*	0	0	
Mixed, *n*	2	0	
Persistent urinary incontinence			1
at 3 months, *n* (%)	0 (0%)	0 (0%)	
at 6 months, *n* (%)	0 (0%)	0 (0%)	
at 12 months, *n* (%)	0 (0%)	0 (0%)	

*IQR* Interquartile Range, *BMI* Body Mass Index, *CCI* Charlson Comorbidity Index, *PSA* Prostate Specific Antigen, *IPSS* International Prostate Symptom Score, *QoL* Quality of life, *Q*max Maximum flow rate, *PVR* Post-void residual, *SHIM* Sexual Health Inventory for Men questionnaire Delta= Baseline – Post-operative parameters difference.

Bold values indicate statistically significant differences between groups based on two-sided testing (*p* 0.05).

Postoperative PSA levels decreased in both groups (0.87 vs. 0.78 ng/mL; *p* = 0.610). Functional outcomes improved significantly in IPSS, QoL, Qmax, and PVR from baseline in both groups, with no significant differences in delta values. SHIM scores remained unchanged postoperatively. TUI was observed in six patients (3.4% LP vs. 7.5% VLP; *p* = 0.272), with no cases of persistent incontinence or urethral stricture (Table 3).

## Discussion

This study evaluated the impact of PV on perioperative, postoperative, and functional outcomes of STEP, with stratification into LP and VLP based on AUA surgical management guidelines [1]. Unlike previous research that primarily focused on PV effects in endoscopic enucleation techniques [17], this is the first study to assess these outcomes in robotic simple prostatectomy using the SP approach. By addressing this gap, our findings provide the first volume-stratified assessment of STEP and support tailored surgical planning for patients with prostates ≥80 cc.

STEP consistently demonstrated favorable and reproducible outcomes across the spectrum of clinically significant PVs. Although enucleation time increased with gland size, rising from a median of 69 min in LP to 84 min in VLP (*p* = 0.002), enucleation efficiency also improved, increasing from 0.97 g/min to 1.23 g/min (*p* = 0.025). These findings suggest that STEP scales proportionally with increasing adenoma burden. Importantly, mucosal flap reconstruction time remained stable between groups, indicating that the added operative time in VLPs was attributable solely to enucleation, not reconstruction, reinforcing the procedure’s efficiency even in VLPs. To account for potential bias from the surgical learning curve, enucleation efficiency was analyzed after adjusting for surgeon and number of prior cases. PV remained the only independent predictor of efficiency (RC = 0.0038 ± 0.0009, *p* < 0.001), whereas experience and the volume–experience interaction were not significant (*p* = 0.46 and *p* = 0.77). Both early (≤median) and late (>median) experience groups demonstrated similar slopes (*p* = 0.78), confirming that STEP’s procedural stability is not experience-dependent but volume-adaptive. The consistent efficiency across the learning curve supports that the transvesical single-incision design inherently facilitates proportional adenoma enucleation without compromising precision, even early in experience.

As PV increases, endoscopic enucleation techniques such as HoLEP, begins to encounter procedural challenges. Although enucleation efficiency improves, morcellation efficiency declines disproportionately, leading to prolonged operative times [18, 19]. In prostates exceeding 200 cc, the bladder may become overfilled with enucleated tissue, compromising visibility and obstructing safe morcellation. Dense, rubbery “beach ball” nodules, which resist standard morcellation, further increase the likelihood of procedural failure [20]. These limitations often necessitate open cystotomy for specimen extraction (up to 17%) or deferment of morcellation to a separate surgical session (up to 17%), both of which compromise the minimally invasive intent of HoLEP. Additional size-dependent constraints, such as limited instrument length and uncontrolled bleeding that may impair visualization, can prompt conversion to OSP (up to 3.4%) [20–24].

Unlike HoLEP, STEP maintains procedural integrity across increasing PVs. Adenoma retrieval is integrated into the enucleation phase, scaling proportionally with gland size due to increased enucleation efficiency and stable reconstruction times. Continuous extraction of enucleated tissue via the robotic arm into an external bubble port (Fig. 1b) eliminates morcellation-related challenges, prevents bladder overdistension, and avoids deferment of adenoma removal to a separate surgical session. Notably, no threshold effect was observed in the VLP group: even in glands exceeding 200 cc, there were no conversions, deferments, or secondary procedures. These results highlight STEP’s adaptability and underscore its procedural stability precisely at the volume threshold where HoLEP’s efficiency begins to decline [21–23].

STEP also demonstrated a strong intraoperative safety profile, with no conversions, no additional port use, and no complications beyond three early cases of venous air embolism (VAE), all of which occurred within the first 28 cases during the initial learning curve. Following protocol refinements, including limiting pneumovesical insufflation pressure to ≤12 mmHg and optimizing intravenous hydration, no further VAEs occurred, and no other intraoperative complications were observed thereafter. Compared with endoscopic enucleation techniques, which report transfusion rates up to 19.3% in prostates >200 cc [21–23, 25], STEP showed a significantly lower transfusion requirement: none in the LP group and only one in the VLP group (PV: 364 cc). This advantage is likely attributable to the consistent use of 360-degree mucosal flap reconstruction, which seals the prostatic fossa, reduces exposed raw surface area, enhances hemostasis, and eliminates the need for CBI [15, 26].

Continence outcomes were similarly favorable, with no cases of persistent UI at three months, in line with prior SP series [27]. In contrast, HoLEP has reported persistent UI rates of up to 23% in VLPs, likely related to the cumulative effects of longer enucleation and morcellation times in larger glands, which prolong energy delivery and intravesical manipulation near the sphincter, predisposing to transient or persistent dysfunction [28–30]. Prior analyses further demonstrate that both enucleation and morcellation times increase with PV, emphasizing that procedural duration itself becomes a volume-dependent source of periurethral strain [18, 31]. MP RASP also appears to yield inferior continence outcomes [4, 7], although volume-stratified data are lacking. STEP’s transvesical approach circumvents these constraints by retrieving the adenoma directly without morcellation, thereby minimizing intravesical manipulation and preserving the integrity of the external sphincter and its supporting structures, which likely contributes to favorable continence recovery [27].

In addition to continence preservation, STEP addresses key limitations of HoLEP and MP RASP, expanding its utility in complex cases. STEP’s suprapubic approach bypasses the urethra entirely, making it feasible in patients with urethral strictures [32] and allowing for simultaneous treatment of bladder stones or diverticula [11]. Compared with MP RASP, which typically requires multiple incisions, steep Trendelenburg positioning, and transperitoneal access under general anesthesia, with associated risks such as postoperative ileus [3], STEP employs a single suprapubic incision and is performed in the supine position, enabling the use of epidural anesthesia [33]. This reduces cardiopulmonary strain, lowers the risk of gastrointestinal complications, and enhances recovery, particularly in elderly or high-risk patients. Furthermore, STEP has demonstrated favorable perioperative outcomes relative to MP RASP, including lower narcotic requirements, reduced complication rates, shorter hospital stay, and earlier catheter removal, highlighting its minimally invasive profile. In patients with a hostile abdomen from prior surgery, STEP also eliminates the need for intra-abdominal dissection [34]. Collectively, these advantages support STEP as a compelling alternative for managing very large prostates, especially in anatomically or physiologically complex patients.

A persistent challenge in BPH surgical management is the inconsistent application of PV thresholds. While AUA and EAU guidelines define prostates ≥80 cc as large, the AUA further defines prostates >150 cc as very large [1, 35]. However, most published series do not adhere to these definitions. RASP studies frequently conflate SP and MP techniques [12], and volume-stratified outcomes are rarely reported. Even extensive HoLEP series inconsistently define LP and VLP categories. In contrast, our study applied standardized PV thresholds and evaluated STEP accordingly, providing a framework for more meaningful volume-dependent comparisons across techniques.

These findings support future head-to-head comparisons between STEP, MP RASP, and HoLEP. Although early series suggest that SP platforms may reduce perioperative morbidity compared with MP approaches, high-quality randomized evidence remains limited, and definitive advantages in functional outcomes or cost-effectiveness have not been established. Prospective and cost-utility studies are needed to clarify the broader clinical and economic role of STEP within the BPH treatment algorithm.

This study has several limitations. It is a retrospective, single-center series from a high-volume institution, which may limit generalizability. Multiple surgeons were involved, although learning-curve analysis showed that enucleation efficiency was driven by PV rather than experience. MRI was the primary modality for PV assessment, with CT or TRUS used selectively when MRI was unavailable or prior imaging existed, which may have introduced minor imprecision near the 150 cc stratification cutoff. The absence of a contemporaneous HoLEP or MP RASP comparator cohort limits direct cross-platform inference. Future multicenter prospective studies with standardized imaging, longer follow-up, and direct comparison groups are required to validate these findings and further define the role of STEP in the management of high-volume BPH.

## Conclusion

This study provides the first volume-stratified analysis of STEP and demonstrates that it maintains procedural efficiency, safety, and excellent functional outcomes across both large (80–150 cc) and very large (>150 cc) prostates. By addressing the morcellation-related and access limitations observed in HoLEP and MP RASP, STEP offers a scalable, minimally invasive alternative that adapts to increasing gland size without compromising performance. Its continuous adenoma retrieval, mucosal flap reconstruction, and supine transvesical access make it particularly advantageous in patients with hostile abdomen, urethral strictures, or a need for concurrent procedures. Our findings support a prostate volume–dependent treatment framework that promotes individualized surgical planning grounded in anatomy and patient-specific factors. Prospective multicenter validation and head-to-head comparisons with existing techniques are warranted to further establish STEP’s role in the surgical management of high-volume BPH.

## Supplementary information

Supplementary Information

## Data Availability

The data associated with the paper is not publicly available, but they are available from the corresponding author on reasonable request.
