# Supplementary material for: Single-port transvesical enucleation of the prostate (STEP) for benign prostatic hyperplasia: a comparative analysis of patients with large (80–150cc) and very large (>150cc) prostate volumes
Source: Prostate Cancer Prostatic Dis. 2026 Feb 21;29(4):791–8. doi: 10.1038/s41391-026-01074-y (PMC13623656; doi:10.1038/s41391-026-01074-y)
Supplement: Supplementary file 1 [file 41391_2026_1074_MOESM1_ESM.docx]

**Supplementary material**

**Figure S1.** Scatterplot Graphs showing specific procedural metrics across the prostate volumes.

**Figure S2.** Baseline and postoperative functional parameters

**Figure S3.** Concurrent procedures alongside STEP; **3a**: Demonstrates the variability of bladder stones and the relative time it takes for cystolithotomy; **3b**: Demonstrates steps of transvesical robotic diverticulectomy.

**Figure S4.** Relationship between prostate volume and case sequence by surgeon for all STEP procedures.

**Figure S5.** Enucleation efficiency as a function of prostate volume in early (≤ median) versus late (> median) surgeon experience.

**Table S1**. Relationship between prostate volume and enucleation efficiency in early vs late experience groups & Interaction test (Early vs Late).

| Experience group | n | Mean PV (cc) | Mean efficiency (g/min) | β per 1 cc (volume → efficiency)* | 95% CI for β | p value |
| --- | --- | --- | --- | --- | --- | --- |
| Early (≤ median cases) | 41 | 164.1 | 1.19 | 0.0050 | 0.0021 - 0.0079 | 0.0016 |
| Late (> median cases) | 41 | 158.1 | 1.34 | 0.0044 | 0.0012 - 0.0076 | 0.0110 |
| *From separate linear regression models of enucleation efficiency on prostate volume within each experience group. | | | | | | |
| Term | | **β difference (Late–Early)** | | | **SE** | **p value** |
| PV × experience group | | −0.00061 | | | 0.00222 | 0.784 |

The positive association between prostate volume and enucleation efficiency was present in both early and late experience groups, with no significant difference in slopes (interaction p = 0.78), indicating that the efficiency–volume relationship was not driven by late-case experience.

**Table S2**. Multivariable models assessing the effect of prostate volume and learning curve on enucleation efficiency

| Model 1: Main adjusted model (no interaction)  Outcome: enucleation efficiency (g/min) | | | | |
| --- | --- | --- | --- | --- |
| term | **β estimate** | **SE** | **t value** | **p value** |
| (Intercept) | 0.0599 | 0.1820 | 0.33 | 0.743 |
| Prostate volume (per cc) | 0.00378 | 0.0009 | 4.20 | <0.001 |
| Prior cases performed | -0.00176 | 0.00236 | -0.74 | 0.460 |
| Surgeon 1 (vs ref) | 0.89157 | 0.21489 | 4.15 | <0.001 |
| Surgeon 2 (vs ref) | 0.07136 | 0.13466 | 0.53 | 0.598 |
|  | | | | |
| Model 2: Interaction model (learning-curve test)  Outcome: enucleation efficiency (g/min) | | | | |
| Term | **β estimate** | **SE** | | **p value** |
| PV × prior cases performed | 0.000019 | 0.000066 | | 0.772 |

Model 1 shows that larger prostate volume is independently associated with higher enucleation efficiency after adjustment for surgeon and case sequence, while prior cases performed is not. Model 2 adds a prostate volume × prior cases interaction term; the non-significant interaction (p = 0.77) indicates that the efficiency–volume relationship does not differ across the learning curve, supporting that the observed increase in efficiency with larger glands is not driven by late-case experience.

**Table S3.** Literature review on HoLEP studies that categorize groups based on prostate volumes for large prostates> 80cc

| Author | Groups | ET | EE | MT | ME | LOS | FCD | BT | Cystost | OC | UI 3M | US | ReTrx |
| --- | --- | --- | --- | --- | --- | --- | --- | --- | --- | --- | --- | --- | --- |
| Boxall et al.  2021 | 80-199  N=157 | **45**  **(40-60)** | **2.1**  **(1.6-2.5)** | **20**  **(15-25)** | 4.9  (3.7-6) | **1 (1-1)** |  | **2 (1.3%)** | **0 (0%)** |  | 9 (5.8%) |  |  |
|  | >200  N=157 | **70**  **(60-90)** | **2.8**  **(2.2-3.5)** | **45**  **(30-60)** | 4.4  (3.2-5.8) | **1 (1-2)** |  | **7 (4.5%)** | **9 (5.7%)** |  | 17 (11%) |  |  |
|  | *p* | **S** | **S** | **S** | NS | **S** |  | **S** | **S** |  | NS |  |  |
| Zell et al.  2021 | 200-299  N=76 | 77.4 ± 28 | **2 ± 0.9** | **47 ± 30** | 4.1 ± 2.5 | 1.9 ± 1.2 | **2.8 ± 2.9** | 16 (21.6%) | 6 (8.1%) | 2 (2.6%) |  |  | 1 (1.3%) |
|  | >300  N=12 | 83 ± 27 | **2.6 ± 1.2** | **75 ± 31** | 2.7 ± 1.4 | 1.7 ± 1.2 | **2.5 ± 2.7** | 1 (8.3%) | 1 (8.3%) | 1 (8.3%) |  |  | 0  (0%) |
|  | *p* | NS | **S** | **S** | NS | NS | **S** | NS | NS | NS |  |  | NS |
| Tay et al.  2022 | <150  N=186 | **59 ± 31** |  | **14 ± 9** |  |  | 1.7 | 0 (0%) |  | 0 (0%) | 29 (16%) | 5 (2.7%) | 1 (0.5%) |
|  | ≥150  N=97 | **86 ± 35** |  | **29 ± 16** |  |  | 2 | 2 (2%) |  | 1 (1%) | 23 (24%) | 1 (1%) | 1 (1%) |
|  | ***p*** | **S** |  | **S** |  |  | NS |  |  |  |  | NS |  |
| Kim et al.  2015 | <100  N=426 | **38 ± 18** | **0.5 ± 0.3** | **10 ± 6** | **1.97 ± 1.2** | **2.3 ± 0.9** | **1.8 ± 2.4** | 0 (0%) | **0**  **(0%)** |  | **25 (6%)** | **0 (0%)** | 0 (0%) |
|  | 100-199  N=70 | **58 ± 18** | **0.9 ± 0.4** | **24 ± 17** | **2.6 ± 1.1** | **2.7± 1.8** | **1.7 ± 1.6** | 0 (0%) | **0**  **(0%)** |  | **3 (4.3%)** | **0 (0%)** | 0 (0%) |
|  | ≥200  N=6 | **77 ± 20** | **1.5 ± 0.8** | **48 ± 28** | **2.2 ± 0.7** | **3.5±1.5** | **4.8 ± 4.8** | 0 (0%) | **1**  **(16.7%)** |  | **2 (33%)** | **1 (16.7%)** | 0 (0%) |
|  | ***p*** | **S** | **S** | **S** | **S** | **S** | **S** | NS | **S** |  | **S** | **S** |  |
| Porto et al.  2023 | ≤40  N=27 | **OT105** |  |  |  | 1 (1) | 1 (1-6) | **0 (0%)** |  |  | 2 (7.4%) | 2 (7.4%) |  |
|  | 41-200  N=332 | **OT150** |  |  |  | 1 (1) | 1 (1) | **4 (1.2%)** |  |  | 58 (18%) | 6 (1.8%) |  |
|  | >200  N=43 | **OT199** |  |  |  | 1 (1) | 1 (1) | **3 (7%)** |  |  | 10 (23%) | 1 (2.3%) |  |
|  | *p* | **S** |  |  |  | NS | NS | **S** |  |  | NS | NS |  |
| ET=Enucleation Time; EE= Enucleation Efficiency; MT= Morcellation Time; ME= Morcellation Efficiency; LOS= Length of Stay; FCD= Foley Catheter Duration; BT= Blood Transfusion; Cystos= Cystotstomy for adenoma retrieval; DR= Delayed retrieval; OC= Open Conversion; UI= Urinary incontinence; UITrx= Surgical intervention for UI; US= Urethral Stricture; ReTrx= Retreatment; S=Significant p<0.05; NS=Not Significant p>0.05 | | | | | | | | | | | | | |
